# Supplementary material for: Learning temporal attention in dynamic graphs with bilinear interactions
Source: PLoS One. 2021 Mar 4;16(3):e0247936. doi: 10.1371/journal.pone.0247936 (PMC7932168; doi:10.1371/journal.pone.0247936)
Supplement: Attachment — Submitted filename: response.pdf [file pone.0247936.s001.pdf]

We thank the reviewers for the useful feedback and comments.
Below is the list of our changes.

Reviewer #1:

1) The task you are focusing on is event prediction, but you don't really state it clearly (casually mentioned in the DyRep backgroup part). Please state it more clearly for clearity.
We added the statement in the beginning of the Experiments section.

2) There is an issue with the figures, they are in the end of the paper instead of embedded in it as I assume the authors intended.
We followed the submission instructions and did not embed the figures in the manuscript.

3) minor typo in dimension, line 205
Fixed.

Reviewer #2:

1. The number of baseline models is too small. It is better to include more results of other models doing dynamic link prediction on the same dataset. In DyREP's paper, Know-Evolve [2], DynGEM [3], GraphSage [4] and GAT [5] are used as baselines. It might strengthen this submission's contributions if the authors could compare one or two more baselines. It might strengthen this submission's contributions if the authors could compare one or two more baselines. Besides, a recent model architecture for link forecasting on dynamic and multi-relational graphs is proposed in [6], which is missing in the related work.
We added the results of GAT and GCN baselines implemented and evaluated using our codebase to Table 3. We also added Table 4 comparing DyRep to Know-Evolve, DynGEM and GraphSage using the results reported in the DyRep paper. We also added a description of our GAT/GCN baselines in Implementation Details and additional discussion of results in Quantitative Evaluation.
We added a paragraph to Related Work discussing [6].

2. To show the superiority of bilinear layers, it is better to do an ablation study regarding it. For example, the authors can first give the experiment result of LDG by maintaining concatenation in equation (8), (10), (12). Then replace these concatenation with bilinear layers in equation (8), (10), (12), and report the experiment result as well.
We added Table 5 with these results.

3. Similar to point 2, the authors can also do another ablation study to show how NRI affects the performance. For example, the authors can first give the experiment result of LDG by maintaining concatenation in equation (8), (10), (12), and then compare this result with the performance of DyREP. To summarize, I recommend this paper to be accepted. However, there is still a lot of work to do to improve it. The most important point is to compare LDG with more models from different angles.
This seems to be very similar to the point above. We hope the added Table 5 together with existing Table 3 provide an evaluation of LDG from different angles.

4. Current datasets contain only thousands of nodes, which might be impractical for real applications. The reviewers understand that the authors mainly aim to compare and beat the DyRep model, therefore, it would be great if the authors could implement and compare their methods on larger datasets, but not necessary.
We agree with this point, but this requires substantial engineering efforts, which we could not allocate in the time limit provided. In general, using our LDG model on larger datasets is possible with more GPU memory and/or more efficient implementation. In this work, we train our models on a single NVIDIA 1080/2080 Ti with 11GB of GPU memory.

[1] DYREP: LEARNING REPRESENTATIONS OVER DYNAMIC GRAPHS
[2] Know-evolve: Deep temporal reasoning for dynamic knowledge graphs
[3] Dyngem: Deep embedding method for dynamic graphs
[4] Inductive representation learning on large graphs
[5] Graph attention networks.
[6] Graph Hawkes Neural Network for Forecasting on Temporal Knowledge Graphs
